# Cooperation between *Lactococcus lactis* NRRL B-50571 and NRRL B-50572 for Aroma Formation in Fermented Milk

**DOI:** 10.3390/foods8120645

**Published:** 2019-12-05

**Authors:** Lilia M. Beltrán-Barrientos, Hugo S. Garcia, Ricardo Reyes-Díaz, María C. Estrada-Montoya, María J. Torres-Llanez, Adrián Hernández-Mendoza, Aarón F. González-Córdova, Belinda Vallejo-Cordoba

**Affiliations:** 1 Unidad de Investigación y Desarrollo en Alimentos (UNIDA), Instituto Tecnológico de Veracruz, Tecnológico Nacional de México, 91897 Veracruz, Mexico; lilia.beltranb@gmail.com (L.M.B.-B.); hsgarcia@itver.edu.mx (H.S.G.); 2 Centro de Investigación en Alimentación y Desarrollo, A.C., 83304 Sonora, Mexicocarmenes@ciad.mx (M.C.E.-M.); mtorres@ciad.mx (M.J.T.-L.); ahernandez@ciad.mx (A.H.-M.); aaronglz@ciad.mx (A.F.G.-C.)

**Keywords:** *Lactococcus lactis*, volatile compounds, aroma, amino acids, proteolysis

## Abstract

The aim of the present study was to characterize the aroma and volatile profiles of milk fermented by wild *Lactococcus lactis* NRRL B-50571 (FM-571) and NRRL B-50572 (FM-572) and co-fermented with both strains (co-FM). Milks fermented by these strains have been reported to have an antihypertensive effect, yet their sensory characteristics, which are of great importance for consumer acceptance of functional foods, have not been studied. In the study, volatiles were determined using solid-phase microextraction gas chromatography mass spectrometry (SPME-GC-MS) and aroma was determined by quantitative descriptive sensory analysis (QDA). Volatile compounds identified in FM-571, FM-572, and co-FM were mainly acids, alcohols, aldehydes, and ketones. FM-571 showed higher total relative volatile abundance than FM-572 or co-FM. Furthermore, the concentrations of specific amino acids (aa) were lower in FM-571 and co-FM than in FM-572. Thus, these results suggested that FM-571 or co-FM are more efficient in transforming specific aa into the corresponding volatiles than FM-572. Indeed, several alcohols and aldehydes, associated with the catabolism of these aa, were found in FM-571 and co-FM, but not in FM-572. Additionally, QDA showed that FM-571 and co-FM presented higher yeasty and cheesy aroma descriptors than FM-572. Also, total aroma intensity scores for FM-571 were higher than those for co-FM or FM-572. Thus, results suggested that the combination of these two specific wild *L. lactis* strains may complement amino acid catabolic routes that resulted in the enhancement or attenuation of aroma production of single strains, presenting new possibilities for the preparation of custom-made starter cultures.

## 1. Introduction

Lactic acid bacteria (LAB) have been used for the production of fermented dairy products as starter cultures, mainly due to their ability to convert lactose to lactic acid [1], and thus provide desirable sensory characteristics. Particularly, *Lactococcus lactis* strains are one of the most important starter cultures used in the dairy industry for the production of cheese, sour cream, and fermented milks [2,3,4]. Also, there has been a rising interest on the development of dairy foods with beneficial health effects [5,6]. In this sense, it has been previously reported that milk fermented with wild *Lactococcus (L.) lactis* NRRL B-50571 or NRRL B-50572 or co-fermented with both strains NRRL B-50571/NRRL B-50572 (co-FM) presented inhibition of the angiotensin converting enzyme activity in vitro (IC_50_ values of 17.62 ± 0.95 µg/mL, 15.53 ± 5.04 µg/mL, and 18.72 ± 1.99 μg/mL, respectively; which is the peptide content necessary to inhibit ACE activity by 50%) [7,8]. Moreover, these fermented milks, when administered to spontaneously hypertensive rats, had antihypertensive, hypolipidemic, and heart rate lowering effects [9,10]. Furthermore, these beneficial effects were attributed to bioactive peptides produced through milk proteolysis during fermentation [11]. Nevertheless, peptides and amino acids derived from milk proteolysis not only may have an impact on bioactivity, but may also contribute to the formation of aroma compounds [12].

During milk fermentation, characteristic aroma compounds may develop by two processes: first, the precursor molecule generates as a result of the breakdown of milk protein, fat and lactose; and then, the transformation of the precursor molecule into the volatile organic aroma compound takes place [13,14]. In dairy products, the breakdown of protein plays an important role in the formation of volatile compounds such as carboxylic acids, aldehydes, ketones, alcohols, and esters that may be generated from amino acid catabolism [15,16]. These volatile compounds provide the characteristic aroma profiles of dairy products and therefore they are the main determining factors of product sensory quality [17].

In this sense, previous studies have reported that wild *L. lactis* isolated from artisanal dairy products may produce larger amounts of volatile compounds and thus different aroma profiles than those present in commercial starters cultures [18]. Furthermore, the combination of lactococcal strains may enhance flavor formation by completion of metabolic pathways [15]. In fact, it has been reported that wild-type lactococcal strains seem to contain more active amino acid-converting enzymes than starter strains, leading to new aromas. Thus, the combination of wild strains can also contribute to aroma diversification [15,19]. On the other hand, several studies have showed that the sensory characteristics are of great importance for consumer acceptance of functional foods [5]. Therefore, it is important to characterize the aroma and volatile profiles imparted by wild *L. lactis* strains before they are used in the development of fermented dairy products with desirable sensory characteristics and beneficial health effects.

Although several studies were carried out to evaluate the potential beneficial effects on health of milks fermented with wild *L. lactis* NRRL B-50571 and NRRL B-50572 or their mixture, as antihypertensive functional foods [7,8,9,10,11], the aroma and volatile compounds that characterize these foods have not yet been assessed. Hence, the objective of the present study was to characterize the aroma and volatile profiles in milks fermented with *L. lactis* NRRL B-50571 and NRRL B-50572 and co-fermented with both strains (1:1, NRRL B-50571:NRRL B-50572).

## 2. Materials and Methods

### 2.1. Strains and Growth Conditions

*L. lactis* strains NRRL B-50571 and NRRL B-50572 were propagated as previously reported [7], in 10 mL M17 broth with sterile lactose (10%, *w*/*v*) (DIFCO, Sparks, MD, USA) and incubated at 30 °C for 24 h. Precultures were obtained by repeating the same procedure twice to allow growth until reaching 10^7^ cfu/mL. A fresh working culture was prepared by inoculating (3%) in sterile (110 °C, 10 min) reconstituted nonfat dry milk (10%, *w*/*w*) and incubated at 30 °C for 12 h, until reaching 10^9^ cfu/mL.

### 2.2. Sample Preparation

Fermented milk with wild *L. lactis* NRRL B-50571 (FM-571) and NRRL B-50572 (FM-572) isolated from artisanal dairy products was prepared as previously reported [11]. Reconstituted (10%, *w*/*v*) dried skim milk was pasteurized (80 °C for 30 min), inoculated with 3% (*v*/*v*) working culture and fermented at 30 °C, until reaching 10^9^ cfu/mL at 48 h. For the co-fermentation with the two strains (*L. lactis* NRRL B-50571 and NRRL B-50572 (co-FM)), milk was inoculated with 1.5% (*v*/*v*) of each working culture (in the ratio 1:1; a total of 3%), and then fermented at 30 °C, until reaching 10^8^ cfu/mL at 48 h. To inactivate *L. lactis*, fermentation was stopped by applying heat treatment (75 °C, 15 min), followed by quick cooling. Unfermented milk was prepared similarly, but without *L. lactis* inoculation. Samples were stored at −20 °C for further volatile analysis.

### 2.3. Microbial Growth Monitoring and pH Determination

Total cell concentrations were determined at 0, 12, and 48 h of fermentation by counts on plates of M17 agar. During fermentation, pH measurements were taken at 12 and 48 h, using a HI 2211 pH and ORP benchtop meter (Hanna Instruments, Woonocket, RI, USA). Measurements were taken in duplicate.

### 2.4. Proteolytic Activity in Fermented Milk

Proteolytic activity in fermented milk was determined by measuring the release of free NH_3_ groups following the o-phthaldialdehyde (OPA) method [20]. An aliquot of 2.5 mL from each fermented milk was mixed with 1 mL H_2_O and 5 mL of 0.75 N trichloroacetic acid (TCA), vortexed for 1 min and rested for 10 min. Afterwards, samples were centrifuged (4696× *g*, 40 min, 4 °C) (ThermoScientific, Chelmsford, MA, USA) and supernatants were filtered with Whatman™ No. 2 paper (8 µm pore size). All filtrates were frozen (−20 °C) until further analysis. A 60 μL sample aliquot containing TCA-soluble peptides were added to 600 μL of fluoraldehyde OPA reagent solution (ThermoScientific, Rockford, IL, USA). After 2 min rest at 20 °C, fluorescence was measured at 360 nm excitation and 455 nm emission (SpectraMax, M3, Sunnyvale, CA, USA). Proteolytic activity was expressed as mg/mL of L-leucine released during milk fermentation, using a standard curve of L-leucine (0–0.125 mg/mL). Unfermented milk was used as control and L-leucine value was subtracted from the fermented milks values. All analyses were carried out in triplicate.

### 2.5. Determination of Free Amino Acids

Free amino acids in fermented milk samples were determined by reversed-phase liquid chromatography (RP-HPLC) (Agilent Technologies 1260 Infinity, Germany) [21]. Briefly, aqueous extracts (<3 kDa) from fermented milk samples were derivatized with fluoraldehyde OPA reagent solution. After derivatization, 10 μL of each sample was injected on a Zorbax 300Extend C18 column, 5 μm, 250 × 4.6 mm (Agilent Technologies, Germany), with detection at 254 nm. Mobile phase A was 100 mM C_2_H_3_NaO_2_, adjusted to pH 7.2 with 1 M HCl, while mobile phase B was methanol (100%). Separation was carried out at a flow rate of 0.75 mL/min with a gradient of 0–5 min with 80% A; followed by 5–8 min with 70% A and 8–15 min with 50% A; and finally, 15–25 min with 20% A. Quantification of free amino acids was calculated with a standard curve constructed with a pool of amino acid standards (0–50 mg/mL).

### 2.6. Solid-Phase Microextraction Gas Chromatography Mass Spectrometry (SPME-GC-MS) Analysis

Headspace solid-phase microextraction and gas chromatography (HS-SPME-GC) was used to identify the volatile compounds present in unfermented milk and fermented milks, following the methodology of González-Córdova and Vallejo-Cordoba [22]. Briefly, samples (9 mL) containing 2.8 g NaCl and 5 μL of an internal standard (nonanoic acid ethyl ester, PolyScience Co., Niles, IL, USA) were placed in 50 mL vials and sealed with headspace vial caps (18 mm magnetic PTFE/Sil, Agilent Technologies). Vials containing the samples were placed on the GC Sampler 80 (Agilent Technologies), and were allowed to equilibrate at 70 °C (30 min, 150 rpm); then, a SPME fiber (CAR-PDMS 75 μm, Supelco Co, Bellefonte, PA, USA) was exposed to the sample headspace (70 °C, 60 min, 150 rpm).

Volatile compounds were determined with a 7890/5975C GC-MS system (Agilent Technologies). The injector was fitted with an inlet liner (78.5 mm × 6.5 mm O.D. × 0.75 mm I.D., Sigma Aldrich^®^ Co. LLC) special for SPME. A high-polarity DB^®^-WAX column (6 m length, 0.25 mm I.D., 0.25 μm film thickness; Agilent J&W Scientific, Santa Clara, CA, USA) was used to separate volatile compounds. After the fiber was charged, it was placed in the injection GC-MS port, and desorbed at 250 °C for 10 min in splitless mode. Helium was used as the carrier gas at 1 mL/min. The initial oven temperature was set at 45 °C for 12.5 min, increased to 114 °C at a rate of 4 °C /min for 6 min, and then to 143 °C at a rate of 7 °C /min for 15 min. Finally, the temperature was increased to 165 °C at a rate of 15 °C/min for 45 min. All volatile compounds were identified by comparing their spectra with those from the program NIST MS library (v. 2.0 g., 2011, Gaithersburg, MD, USA). Identification of compounds was also achieved using retention times of standard compounds. Furthermore, relative volatile abundance was calculated by (peak area/area of internal standard x internal standard concentration). The internal standard was nonanoic acid ethyl ester, 1000 mg/L in methanol.

### 2.7. Aroma Sensory Evaluation

For aroma intensity, unfermented and fermented milks were evaluated with quantitative descriptive analysis (QDA) [23]. Samples were assessed for the different aroma descriptors derived using a 15 cm reference scale (0 = absence of aroma; 15 = intense aroma given by a reference sample). A panel of eight trained panelists participated in the aroma evaluation of samples with controlled conditions of lighting and environment. The panel was considered trained when reproducible responses (no statistical differences at alpha = 0.05) among panelists were given for each descriptor.

### 2.8. Statistical Analysis

One-way analysis of variance (ANOVA) was used to evaluate data obtained from sensory and volatile analysis. Differences among means were assessed by Scheffe multiple comparison test and considered significant when *p* ≤ 0.05. Principal component analysis (PCA) was performed to relate the relative abundance (μg/L) for each volatile compound and sensory evaluation data obtained for each descriptor. Clusters were determined by the k-means clustering analysis. Data are presented as means ± SD; using the Stata statistical software (version 11, Statcorp., College Station, TX, USA).

## 3. Results and Discussion

### 3.1. Microbial Growth and Acidifying Activity

Microbial counts of milk cultures were initially 2.79 × 10^7^ cfu/mL and 3.02 × 10^7^ cfu/mL for FM-571 and FM-572, respectively; and reached 1.85 × 10^9^ cfu/mL and 1.91 × 10^9^ cfu/mL, respectively, after 12 h of fermentation. Also, these strains were highly acidifying since pH significantly (*p* < 0.05) decreased from 6.5 ± 0.07 to 4.59 ± 0.04 and 4.57 ± 0.02, respectively, for FM-571 and FM-572, after 12 h of fermentation.

When these working cultures were inoculated to milk to produce FM-571, FM-572, and co-FM, microbial counts were (0 h) 4.57 × 10^7^ cfu/mL, 3.05 × 10^7^ cfu/mL, and 3.15 × 10^7^ cfu/mL, respectively. Then, after 48 h of fermentation, total cell concentrations significantly (*p* < 0.05) increased to 1.85 × 10^9^ cfu/mL and 1.91 × 10^9^ cfu/mL for FM-571 and FM-572, respectively. Furthermore, for co-FM total cell concentration significantly (*p* < 0.05) increased to 6.7 × 10^8^ cfu/mL. Nevertheless, single cell concentration in co-FM was not evaluated, since it would not be possible to distinguish colonies for the individual strains by total plate counts. It has been well established that the interaction of starter cultures may act as synergistic or antagonistic. Thus, since total cell concentration was one log lower in co-FM than in single fermentations, an antagonistic interaction may be taking place in co-FM [24]. However, further studies are required in order to understand the interaction of these two strains during microbial growth. It has been reported that antagonism may be based on the production of substances that inhibit or by inactive bacteria, such as bacteriocins, and other compounds [24,25].

Additionally, pH significantly (*p* < 0.05) decreased after 48 h of fermentation reaching 4.31 ± 0.05, 4.3 ± 0.06, and 4.02 ± 0.03 for FM-571, FM-572, and co-FM, respectively. Thus, both strains were highly acidifying. Furthermore, their co-culture in co-FM presented a significantly (*p* < 0.05) lower pH. It has been reported that wild *L. lactis* strains isolated from dairy products presented high acidifying activity; furthermore, this was not only strain-dependent but it was also determined by the source of isolation [26]. Milk is a rich source of nutrients, such as lactose and its utilization by LAB determines lactic acid production [27]. Nevertheless, competition and cooperation between LAB occurs for several metabolites with different mechanisms involved [28].

### 3.2. Milk Proteolysis and Free Amino Acid Determination

**Figure 1:**
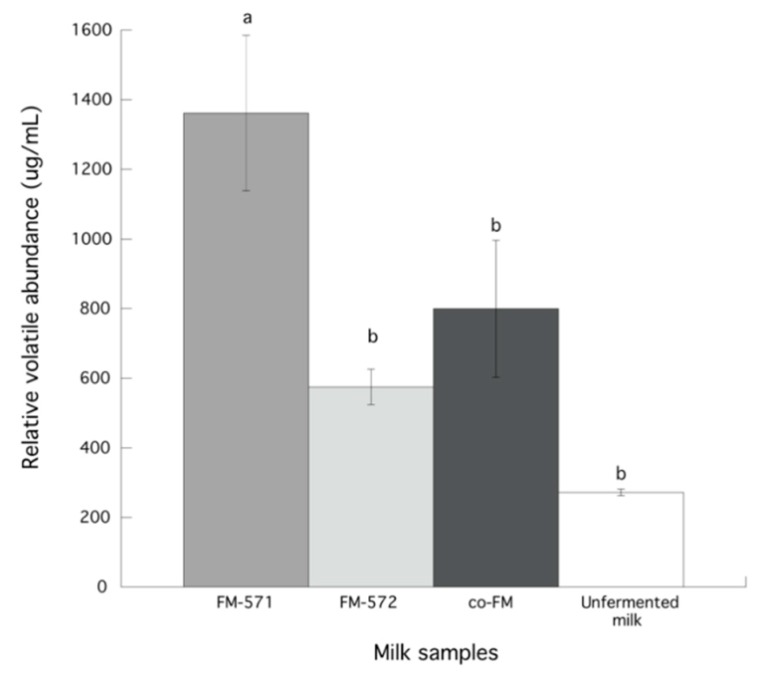
Total relative volatile abundance (μg/L) in unfermented and fermented milks. Data are presented as means ± SD. Data with different letter are significantly different (*p* < 0.05).

It has been reported that high proteolytic activity in fermented dairy products, delivers a greater production of volatile compounds [29], thus, in this study proteolytic activity of two wild *L. lactis* strains was determined in the fermented milks. Fermented milk with both strains (co-FM) presented significantly (*p* < 0.05) higher proteolytic activity (131.53 ± 32.04 μg/mL L-leucine) than FM-572 (37.64 ± 1.06 μg/mL L-leucine), FM-571 (23.81 ± 0.67 μg/mL L-leucine), or unfermented milk (17.56 ± 0.64 μg/mL L-leucine). Nevertheless, FM-571 showed significantly larger (*p* < 0.05) total relative volatile abundance (μg/L) than FM-572 or co-FM (Figure 1). Thus, it appears that although co-FM or FM-572 presented higher proteolytic activity than FM-571 that may favor the formation of more flavor precursor molecules (amino acids), the determining factor for volatile production may be the transformation of amino acids into volatile compounds [13,14]. Although total cell concentration was one log cycle lower for co-FM (*p* < 0.05) than for the single strains, higher proteolytic activity in co-FM, may be the result of metabolic cooperation between both strains. It has been previously reported that combinations of LAB with high proteolytic activities are some of the most common tools used to accelerate and diversify proteolysis during dairy fermentations [28].

It has been reported that the transformation of amino acids to volatile compounds by *Lactococcus lactis* begins with the production of α-ketoacids by transamination reactions where several enzymes are involved [30,31]. In this sense, during milk fermentation, peptides and amino acids are released from milk proteins by the LAB proteolytic system, since these compounds are essential for microbial growth in milk [32] Therefore, although FM-571 may present fewer free amino acids, due to a relatively weaker proteolytic system [30] in this strain than that in FM-572, the transformation of amino acids into volatile compounds may be more efficient in FM-571.

Furthermore, amino acids resulting from the proteolytic activity for each LAB, may be the main precursors for a complex series of catabolic reactions that produce important specific aroma compounds, such as alcohols, aldehydes, esters, and acids [33]. In this study, free amino acid profiles showed marked differences among the aqueous extracts (<3 kDa) of these fermented milks. Furthermore, amino acid concentrations showed that L, I, V, and E were significantly lower (*p* < 0.05) in the aqueous extracts (<3 kDa) from FM-571 or co-FM than in those from FM-572 (Table 1). Thus, these results suggested that *L. lactis* in FM-571 and *L. lactis* in co-FM were more efficient in transforming these amino acids into the corresponding volatiles than those in FM-572.

Indeed, 3-methyl-butanal and 3-methyl butanol, that were reported to be derived from leucine [14], were present in FM-571 and co-FM, but not in FM-572 (Table 2). Also, 3-methyl butanoic acid content, that was reported to be derived from leucine [14], was found in all fermented milks but was significantly (*p* < 0.05) higher in FM-572. Thus, it appears that in this sample, 3-methyl butanal was further transformed to the corresponding acid (3-methyl butanoic acid). Similarly, 2-methyl propanol, that was reported to be derived from valine [14], was only identified in FM-571 (Table 2). Furthermore, significantly (*p* < 0.05) lower glutamic acid content was observed in FM-571 than in FM-572 (Table 1), that may be related to a higher total volatile relative abundance in FM-571 (Figure 1). It has been reported that glutamate participates in the formation of α-ketoglutarate which provides precursors for the catabolism of other amino acids [34]. Thus, it appears that lower glutamic acid content in FM-571 may be related to a higher transformation of other amino acids into volatile compounds.

The aromatic amino acid, phenylalanine (F) was significantly lower (*p* < 0.05) in the aqueous extracts (<3 kDa) from FM-571 or co-FM than in that from FM-572 (Table 1). In fact, phenyl acetaldehyde and phenylethyl alcohol, that were reported to be derived from F [13], were present in FM-571 and co-FM, but not in FM-572 (Table 2).

In general, although FM-571, showed the least milk proteolysis, it presented the largest total relative volatile abundance, probably attributed to a higher amino acid transformation to volatile compounds [35]. Furthermore, the co-fermentation of *L. lactis* NRRL B-50571 and NRRL B-50572 in co-FM, may increase some specific volatiles that may be desirable for fermented milk, but may also attenuate others, such as 3-methyl butanol, that may have a pronounced influence on a cheesy aroma that may not be desirable in fermented milk (Table 2). Indeed, it has been reported that 3-methyl butanol imparts a strong cheese aroma [35].

### 3.3. Identification and Chemical Groups of Volatile Compounds

**Figure 2:**
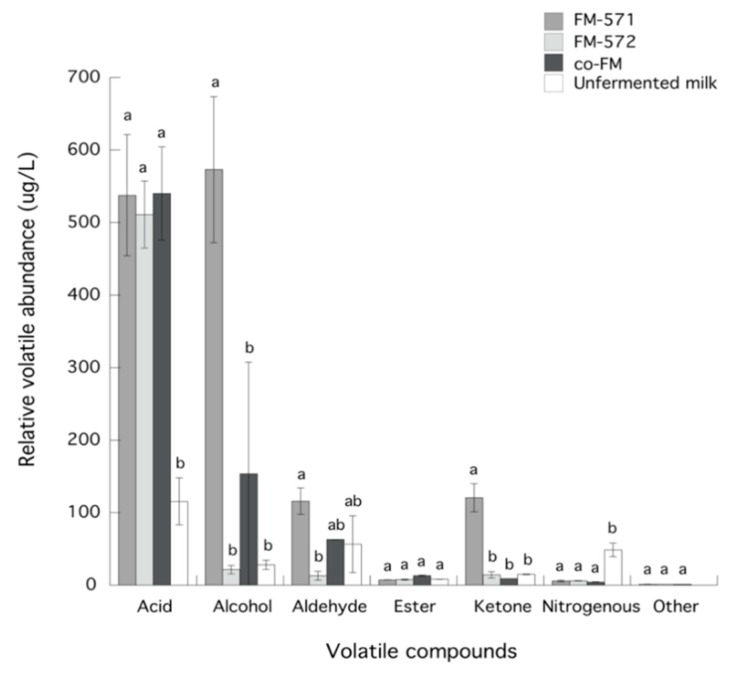
Relative volatile abundance of chemical groups of compounds in unfermented and fermented milks. Data are presented as means ± SD. Data for each chemical group sharing the same letter are not significantly different (*p* > 0.05).

Volatile compounds and their relative abundance (μg/L) in fermented and unfermented milks are presented in Table 2. Results showed 26, 17, and 22 volatile compounds present in FM-571, FM-572, and co-FM, respectively. Thus, to gain some insight into the nature of the differences in these compounds for the tested milks, total relative abundance for each chemical group of volatile compounds is depicted in Figure 2. In general, acids were the group with the greatest relative abundance for all fermented milks and were not significantly (*p* > 0.05) different among them. Also, FM-571 showed significantly (*p* < 0.05) higher relative abundance for alcohols, aldehydes, and ketones than FM 572 and co-FM (Figure 2). During milk fermentation, volatile compounds may be formed mainly from microbial transformations, although enzymatic or chemical transformations of lactose, lipids, citric acid, and proteins may also take place. In many cases, the differences between aroma and volatile profiles from fermented dairy products, has been directly attributed to the presence of different bacterial strains [16].

A total of seven volatile acid compounds were detected in all fermented milks. Acetic acid was significantly (*p* < 0.05) higher in fermented milks than in unfermented milk (Table 2). It has been reported that acetic acid gives a pungent, acidic, sharp vinegar-like aroma to milk [17,36,37]. Acetic acid has been previously identified in unfermented milk samples [38,39] and in several fermented milks, such as a milk fermented with *Lactobacillus pentosus* (15), *Lactobacillus casei* DN-114 001 [38], *Streptococcus thermophilus* IMAU80842 and *Lactobacillus delbrueckii* ssp. *bulgaricus* IMAU20401 [40], *Lactobacillus casei* GBHM-21 [37], and *Streptococcus thermophilus* MGA45-4 [17].

Also, hexanoic and octanoic acids were significantly (*p* < 0.05) higher in all fermented milks than in unfermented milk. Moreover, hexanoic compound was also the most abundant in FM-572 or in co-FM. This volatile compound has also been reported in several dairy products [16,37,41], and it provides a sour, fatty, sweaty, cheesy flavor to dairy products [39]. Meanwhile, octanoic acid was the most abundant acid compound in FM-571 and the second most abundant in FM-572 and in co-FM. This compound delivers a fatty, waxy, rancid, oily, vegetable and cheesy flavor [42].

Interestingly, 3-methylbutanoic acid was only present in fermented milks. This compound is derived directly from amino acid metabolism (2-methyl-butanal), and it provides a cheesy, sweaty, rancid, fecal, rotten fruit, and goat-like aroma profile [43]. Additionally, butanoic acid was present in all samples. This compound delivers rancid, cheesy, and sharp aroma [44].

Furthermore, heptanoic and n-decanoic acids were also only detected in the fermented milks. Acids may be released by lipolysis and from the degradation of lactose and amino acids, and may be responsible for the rancid flavor in milk [45,46]. Also, acids are precursors for ketones, alcohols, aldehydes, and esters, all of which may impact milk flavor [47].

Alcohol comprised the second most abundant group of compounds in all samples (Figure 2). Five, two, and four volatile alcohols were detected in FM-571, FM-572, and co-FM, respectively (Table 2). Alcohol compounds have also been reported in dairy products, and have a substantial influence on aroma. It has been widely reported that alcohol compounds may be produced from ketones, aldehydes, and/or amino acids [13,17,48].

Two methyl alcohols were identified in fermented milk samples: 2-methyl-1-propanol and 3-methyl-1-butanol. In FM-571 and co-FM, 3-methyl-1-butanol content was the most predominant and was significantly (*p* < 0.05) higher than in unfermented milk. This compound may be derived from the reduction of 3-methyl butanal, by alcohol dehydrogenase [49]. Furthermore, it has been previously reported to provide cheesy aroma in Gouda cheese [50] and fresh cheese aroma to Mozzarella cheese [51]. Secondary alcohols may be derived from methyl ketones by the action of reductases [52]. Moreover, phenylethyl alcohol was also identified only in FM-571 and in co-FM. Amino acids such as L-phenylalanine may also be precursors for alcohols, and may produce phenylethyl alcohol [53]. Additionally, 2-ethyl-1-hexanol was also found in all samples, but was significantly higher (*p* < 0.05) in unfermented milk than in fermented milks. Ethanol was detected in all fermented milks, and relative contents were not significantly different (*p* > 0.05) among them. Ethanol has also been reported in milks fermented with *Streptococcus thermophilus* IMAU80842 and *Lactobacillus delbrueckii* ssp. *bulgaricus* IMAU20401 [40]. Interestingly, ethanol has been associated to yeasty flavor in Cheddar cheese [54]. Although some authors have suggested that ethanol does not provide any aroma in yogurts, other studies proposed that ethanol may deliver a complementary aroma [55].

Aldehydes comprised the third most abundant group of compounds in all samples. A total of six, two, and five aldehydes were identified in FM-571, FM-572, and co-FM, respectively (Table 2). Hexanal was found in unfermented milk and in two of the fermented milks; however, relative content was significantly (*p* < 0.05) higher in unfermented milk. This volatile compound has been reported to have a fresh, green, fatty, aldehydic, grassy, leafy, fruity, and sweaty flavor description [42]. Benzaldehyde was also detected in all samples; however, relative contents were not significantly different (*p* > 0.05) among them. It has been previously reported that this compound may provide a unique aroma profile to fermented milks, since at low levels it gives aroma notes like bitter almonds and at high levels contributes to aroma notes like maraschino cherries [56]. This compound was also identified in other fermented milks [17,40). Other aldehydes such as 3-methyl-2-butenal and phenyl acetaldehyde were also detected in fermented milks. Additionally, 3-methyl-butanal that was detected in FM-571 and co-FM but not in FM-572, was reported to provide cheesy aroma notes [51]; these cheese-like notes may be produced through microbial or enzymatic reactions from amino acids [57]. Generally, aldehyde compounds are short-lived components in milk products as they are rapidly reduced into alcohols or oxidized into acids [49,58]; hence, they are present in rather low concentrations [49].

In total, four, two, and two ketone compounds were detected in FM-571, FM-572, and co-FM, respectively (Table 2). Ketone compounds are formed mostly by the thermal degradation of amino acids, oxidation of free fatty acids, and the Maillard reaction [56]. In dairy products such as cheese, ketones and methyl ketones may provide fruity notes [59].

2-nonanone and 2-heptanone were identified in all samples. The relative contents of 2-heptanone for all samples were not significantly different (*p* > 0.05). However, the relative content for 2-nonanone was significantly (*p* < 0.05) higher for FM-571, FM-572, and co-FM, than for the unfermented milk. Although, in some fermented dairy products, 2-heptanone was related to musty, sweet, moldy, and varnish aromas, and 2-nonanone to floral, fruity, and peachy aroma, other studies have reported that these two compounds are responsible for the “stale” flavor of heated milks [38,60]. Additionally, other ketones such as 2-methyl-3-heptanone and 5-hydroxy-2,7-dimethyl-4-octanone, were also detected in FM-571, but not in the other samples.

In fermented milks, only two esters were detected and 2-methyl-octanoic acid ethyl ester was identified as the most abundant (Table 2). Esters are produced mainly via the esterification of free fatty acids and alcohols [61]. Generally, most esters may provide fruity and floral flavors to fermented milks, thus decreasing the pungent and astringent aroma of fatty acids and amines [55]. Propanoic acid, 2-methyl-, 2-phenylethyl ester was detected at low levels in FM-571. Additionally, limited levels of formic acid and octyl ester were detected in FM-572. Although ester compounds may have low taste thresholds, low levels of esters may still provide positive flavor notes in fermented milk products [41]. The production of ester compounds has been previously reported in lactococci fermented dairy products [62].

Oxime-methoxy phenyl was the only nitrogenous compound identified in all samples. In fact, relative abundances in unfermented milk were significantly (*p* < 0.05) higher than those in the fermented milks. Several authors have reported that this compound was detected in UHT milk samples [38,63] and pasteurized milk samples [46]. Other authors have stated that oximes may be produced by the reaction of aldehydes or ketones with a nitrogen-containing reducing agent in a weakly acidic medium during high heat treatment and/or homogenization [39]. Likewise, other nitrogenous compounds were also reported in fermented milk with *Lactobacillus helveticus* H9 [6]. Lastly, butanimidamide was identified in all fermented milks. Relative abundances were not significantly different (*p* > 0.05) between them. Only one study has previously reported this compound in dulce de leche, a Latin American dairy product [64].

### 3.4. Aroma Sensory Evaluation

**Figure 3:**
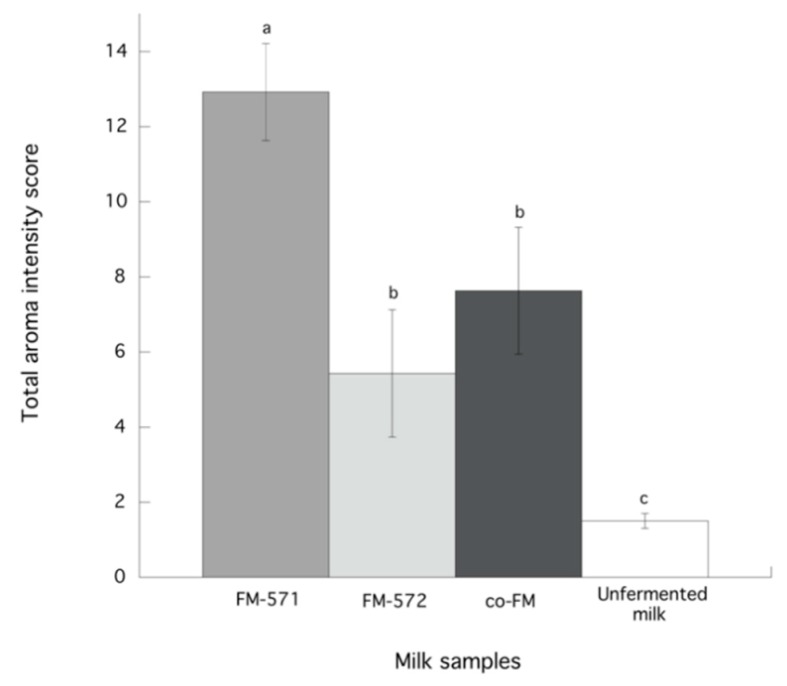
Total aroma scores of unfermented milk and fermented milks. Data are presented as means ± SEM. Data sharing the same letter are not significantly different (*p* > 0.05).

**Figure 4:**
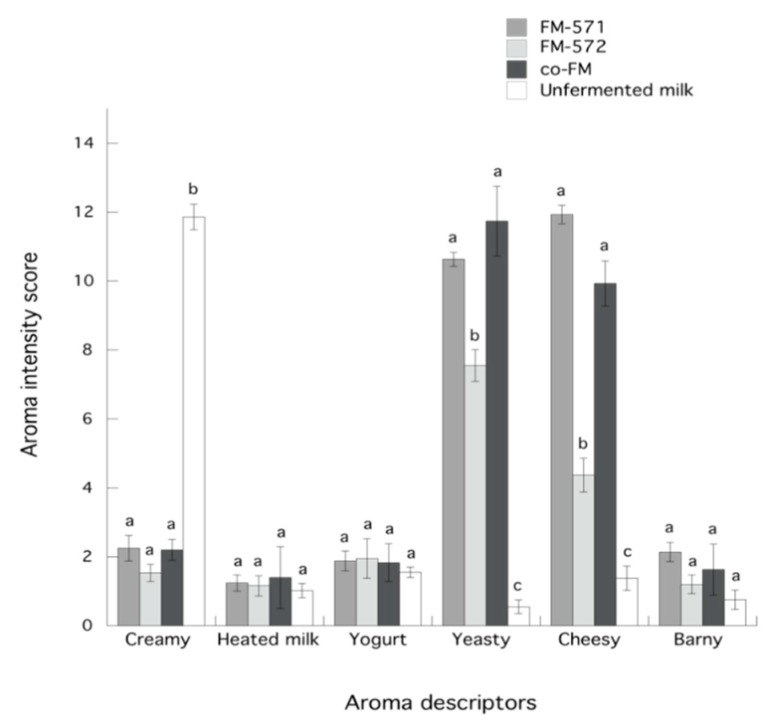
Aroma scores for descriptors in unfermented milk and fermented milks. Data are presented as means ± SEM. Data between aroma descriptors sharing the same letter are not significantly different (*p* > 0.05).

After describing the aroma profile of fermented and unfermented milks, specific aroma descriptors were defined according to notes perceived by the panelists (Table 3). Additionally, total aroma scores for fermented and unfermented milks were calculated (Figure 3). Data showed that the total intensity score for FM-571 was significantly (*p* < 0.05) higher than those for the other fermented milks. Indeed, this higher aroma score may be related to the fact that FM-571 also presented the higher total relative volatile abundance of all fermented milks (Figure 1). Also, results indicated no significant differences (*p* > 0.05) for aroma descriptors such as heated milk, yogurt, and barny among all samples (Figure 4). As expected, the creamy aroma descriptor was significantly (*p* < 0.05) higher for unfermented milk than for fermented milks. However, for yeasty and cheesy aroma descriptors, scores were significantly (*p* < 0.05) higher in the fermented milks than in the unfermented milk (Figure 4). In this sense, yeasty aroma presented significantly (*p* < 0.05) higher scores for FM-571 and co-FM than for FM-572. This aroma descriptor has been previously related to ethanol in Cheddar cheese.

Additionally, the cheesy aroma descriptor was significantly (*p* < 0.05) higher for FM-571 and co-FM than for FM-572. It has been previously reported that compounds such as 3-methyl butanal, 3-methyl butanol, and phenyl ethyl alcohol highly correlated with ripened cheese aroma [19]; hence, these intense cheesy notes may be attributed to the higher relative contents of these compounds in FM-571 and co-FM (Table 2).

**Figure 5:**
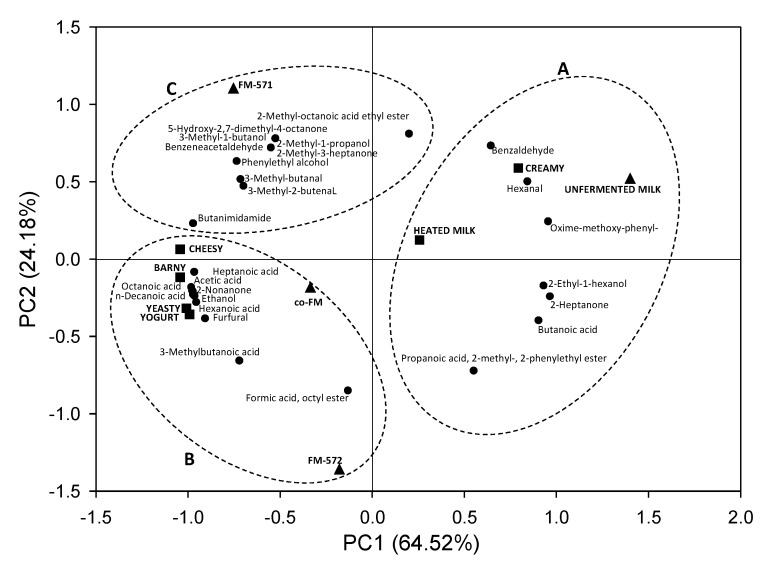
Principal component analysis (PCA) of the first two principal components of volatile compounds, aroma descriptors and samples. A, B, and C: Groups determined by k-means cluster analysis.

Principal component analysis was performed in order to interpret how the volatile and aroma descriptors are associated with the samples (Figure 5). The first principal component (PC1) explained 64.52% of the total variation and PC2 explained 24.18%. Altogether, PC1 and PC2 accounted for 88.7% of the total data variation. Three main groups were identified: group A contained unfermented milk, group B contained FM-572 and co-FM, and group C, contained FM-571. PCA of the volatiles present also showed a clear distinction among sample groups (Figure 5).

Group A, where the unfermented milk was located, clustered aroma volatiles (benzaldehyde, hexanal, oxime-methoxy-phenyl, 2-ethyl-1 hexanone, 2 heptanone, butanoic acid, propionic acid, 2-methyl-, and 2-phenyl ethyl ester) were associated to creamy and heated milk descriptors. In fact, some of these compounds were previously reported to be associated to heated milk [39,46]. On the other hand, hexanal was reported to have a fatty, aldehydic, grassy, leafy, and sweaty aroma descriptor [42], and was also positioned near the creamy descriptor.

Groups B and C, where all fermented milks were located, clustered aroma volatiles associated to cheesy, yeasty, barny, and yogurt aroma descriptors. In fact, closeness of specific compounds (3-methyl-butanal, 3-methyl butanoic acid, and 3-methyl-butanol) to the cheesy aroma descriptor confirms the fact that these compounds were reported to be associated to cheese aroma [19].

Although, FM-571 and FM-572 were located far apart in PC2 in groups C and B, in PC1 these two samples were not distant. Interestingly, co-FM was located in group B, almost equidistant to FM-571 and FM-572, where more volatiles were clustered. FM-571, that was the sample that presented the highest aroma score (12.3) (Figure 3), was located in group C where 10 volatile compounds were clustered (Figure 5). Also, co-FM, with an aroma intensity score of 7.6 (Figure 3) was located in group B, where FM-572 was located together with 10 volatiles (Figure 5). In fact, FM-572 with an aroma intensity score of 5.4 (Figure 3), was not significantly (*p* < 0.05) different from co-FM with an intensity aroma score of 7.6 (Figure 3). Thus, PCA was able to graphically confirm the association of specific volatile compounds with aroma descriptors and milk samples.

## 4. Conclusions

To the best of our knowledge, this is the first study which evaluates aroma and volatile profiles of antihypertensive fermented milks. Volatile compounds deliver characteristic aroma notes that are strain-dependent. Thus, these specific starter cultures are not only adequate for the production of fermented milks with antihypertensive properties, but they also may deliver characteristic and desirable aroma profiles. Also, the combination of these specific wild *L. lactis* strains may complement catabolic routes that may result in the enhancement or attenuation of aroma formation of single strains, thus presenting new possibilities for the preparation of custom-made starter cultures. Further studies are required in order to understand how these two strains interact to complement their metabolisms during milk fermentation.

## Figures and Tables

**Table 1:** Free amino acid quantification (μg/mL) in aqueous extracts (<3 kDa) from fermented milks.

Amino Acid (μg/mL)		FM-571	FM-572	Co-FM
Aspartic acid	N	0.67 ± 0.17 ^a^	0.2 ± 0.01 ^b^	0.26 ± 0.07 ^b^
Glutamic acid	E	11.06 ± 0.50 ^a^	142 ± 1.3 ^b^	13.83 ± 0.53 ^c^
Serine	S	2.95 ± 0.19 ^a^	3.93 ± 0.24 ^b^	3.69 ± 0.5 ^b^
Histidine	H	14.33 ± 0.34 ^a^	21.09 ± 0.42 ^b^	18.94 ± 0.29 ^c^
Arginine	R	9.03 ± 0.78 ^a^	8.09 ± 0.14 ^a^	12.39 ± 0.19 ^b^
Threonine	T	10.10 ± 0.15 ^a^	10.57 ± 0.10 ^a^	10.21 ± 0.28 ^a^
Glycine	G	5.51 ± 0.21 ^a^	4.13 ± 0.14 ^b^	5.27 ± 0.82 ^ab^
Tyrosine	Y	38.86 ± 2.68 ^a^	17.69 ± 0.14 ^b^	38.92 ± 0.02 ^a^
Alanine	A	58.68 ± 1.07 ^a^	12.88 ± 0.11 ^b^	68.36 ± 0.37 ^c^
Tryptophan	W	0.36 ± 0.09 ^a^	0.47 ± 0.10 ^a^	0.87 ± 0.08 ^b^
Methionine	M	1.69 ± 0.08 ^a^	0.91 ± 0.10 ^b^	3.78 ± 0.23 ^c^
Valine	V	ND	3.97 ± 0.24 ^a^	1.73 ± 0.05 ^b^
Phenylalanine	F	5.84 ± 0.39 ^a^	23.30 ± 0.39 ^b^	12.56 ± 0.23 ^c^
Isoleucine	I	0.97 ± 0.02 ^a^	5.89 ± 0.68 ^b^	0.19 ± 0.03 ^a^
Leucine	L	3.34 ± 0.10 ^a^	15.30 ± 0.32 ^b^	2.85 ± 0.04 ^a^
Lysine	K	13.87 ± 0.33 ^a^	22.52 ± 0.41 ^b^	17.11 ± 0.78 ^c^
Asparagine	N	ND	ND	ND
Glutamine	Q	ND	ND	ND

Data for each amino acid between fermented milk samples sharing the same letter are not significantly different (*p* > 0.05). ND: not detected.

**Table 2:** Relative abundance (μg/L) of volatile compounds present in unfermented and fermented milks.

Volatile Compound	RT ^1^ (min)	Relative Abundance (μg/L)			
		Unfermented Milk	FM-571	FM-572	Co-FM
Acids					
Acetic acid **	37.92	4.09 ± 2.30 ^a^	90.53 ± 12.97 ^b^	69.39 ± 24.79 ^b^	72.25 ± 58.92 ^b^
Butanoic acid **	45.35	45.81 ± 31.22 ^a^	24.98 ± 3.64 ^a^	36.12 ± 15.76 ^a^	33.84 ± 7.03 ^a^
3-Methylbutanoic acid *	47.71	ND	2.88 ± 0.58 ^a^	7.19 ± 6.86 ^a^	3.06 ± 1.94 ^a^
Hexanoic acid **	58.68	34.08 ± 2.45 ^a^	163.96 ± 26.07 ^b^	171.75 ± 39.22 ^b^	186.28 ± 1.52 ^b^
Heptanoic acid **	64.89	ND	2.25 ± 0.48 ^a^	1.89 ± 0.12 ^a^	2.44 ± 0.26 ^a^
Octanoic acid **	73.23	31.47 ± 3.58 ^a^	184.32 ± 26.40 ^b^	164.50 ± 6.59 ^b^	178.31 ± 7.81 ^b^
*n*-Decanoic acid **	102.49	ND	68.79 ± 13.33 ^a^	60.12 ± 2.72 ^a^	63.85 ± 4.01 ^a^
Alcohols					
Ethanol **	11.19	ND	16.73 ± 5.21 ^a^	15.62 ± 2.11 ^a^	20.02 ± 7.57 ^a^
2-Methyl-1-propanol *	20.28	ND	2.92 ± 0.73	ND	ND
3-Methyl-1-butanol *	26.85	5.37 ± 5.76 ^a^	273.14 ± 37.52 ^b^	ND	76.41 ± 104.76 ^ab^
2-Ethyl-1-hexanol *	39.44	22.69 ± 0.65 ^a^	2.57 ± 0.44 ^b^	6.00 ± 3.72 ^b^	8.02 ± 4.87 ^b^
Phenylethyl alcohol **	56.70	ND	277.73 ± 58.84 ^a^	ND	49.07 ± 62.68 ^a^
Aldehydes					
3-Methyl-butanal *	10.20	ND	41.95 ± 8.50 ^a^	ND	44.28 ± 5.21 ^a^
Hexanal **	19.52	23.71 ± 7.37 ^a^	2.52 ± 1.61 ^b^	ND	2.94 ± 2.06 ^b^
3-Methyl-2-butenal *	25.28	ND	1.88 ± 0.16 ^a^	ND	2.05 ± 0.29 ^a^
Furfural **	38.38	1.58 ± 0.04 ^a^	2.84 ± 0.75 ^a^	3.18 ± 0.23 ^a^	2.63 ± 0.36 ^a^
Benzaldehyde **	40.99	31.19 ± 31.55 ^a^	20.07 ± 3.69 ^a^	9.95 ± 6.29 ^a^	11.09 ± 6.99 ^a^
Phenyl acetaldehyde *	46.31	ND	46.60 ± 6.75	ND	ND
Ketones					
2-Methyl-3-heptanone *	23.15	ND	34.34 ± 7.74	ND	ND
2-Heptanone *	24.78	13.42 ± 0.16 ^a^	7.10 ± 0.07 ^a^	9.61 ± 4.33 ^a^	9.05 ± 1.01 ^a^
2-Nonanone *	30.45	1.52 ± 0.25 ^a^	4.95 ± 0.52 ^b^	4.68 ± 0.20 ^b^	4.18 ± 0.30 ^b^
5-Hydroxy-2,7-dimethyl-4-octanone *	39.44	ND	74.29 ± 11.21	ND	ND
Esters					
2-Methyl-octanoic acid ethyl ester *	36.36	8.21 ± 0.08 ^a^	6.63 ± 0.05 ^b^	0.94 ± 0.38 ^b^	8.14 ± 0.17 ^a^
Formic acid, octyl ester *	42.18	ND	ND	1.72 ± 0.70 ^a^	0.99 ± 0.10 ^a^
Propanoic acid, 2-methyl-, 2-phenylethyl ester *	65.59	ND	0.68 ± 0.08	ND	ND
Nitrogenous					
Oxime-methoxy-phenyl- *	53.02	48.70 ± 9.45 ^a^	5.83 ± 1.19 ^b^	6.04 ± 0.27 ^b^	4.34 ± 0.33 ^b^
Other compounds					
Butanimidamide *	44.02	ND	1.29 ± 0.23 ^a^	1.14 ± 0.05 ^a^	1.20 ± 0.03 ^a^

^1^ Retention time; ND: not detected. Data for each volatile compound between milk samples sharing the same letter are not significantly different (*p* > 0.05). *: Volatile compound identified by GC-MS only. **: Volatile compound identified by both GC retention time of a standard compound and GC-MS.

**Table 3:** Selected aroma descriptors for sensory evaluation.

Aroma Descriptors	Reference Sample
Creamy	Heavy cream
Yogurt	Unflavored yogurt
Yeasty	Raw yeast dough
Cheesy	Commercial raw milk Mexican Fresco cheese
Barny	Stable hay
Heated milk	Condensed milk
